# The Effect of Humic Acid and Polystyrene Fluorescence Nanoplastics on *Solanum lycopersicum* Environmental Behavior and Phytotoxicity

**DOI:** 10.3390/plants11213000

**Published:** 2022-11-07

**Authors:** Dhivya Lakshmikanthan, Natarajan Chandrasekaran

**Affiliations:** Centre for Nanobiotechnology, Vellore Institute of Technology, Vellore 632014, India

**Keywords:** chlorophyll estimation, humic acid (HA), oxidative stress, fluorescence polystyrene (Flu−PS), seed germination

## Abstract

The impacts of nanoplastics (100 nm) on terrestrial systems are unclear at this time. Due to the utilization of sewage sludge, plastic particles are likely to accumulate in these systems. The current research investigates how *Solanum lycopersicum* seed germination and growth are affected by fluorescence polystyrene (Flu−PS), humic acid (HA), and a Flu−PS+HA combination (tomato). Following 24 h of interaction between Flu−PS and HA, our report details the development of an eco-corona with a significant increase in hydrodynamic size. Plant growth, seed germination, and chlorophyll content were all enhanced by the eco-coronated Flu−PS.Additionally, we discover that seeds treated with Flu−PS+HA demonstrated a germination rate of 90%, compared to just 65.8% for seeds treated with Flu−PS alone. Chlorophyll (a, b, and a + b) content measurements indicated that HA-treated groups and Flu−PS+HA-treated groups had considerably higher levels of chlorophyll (a, b, and a + b) than Flu−PS-treated groups (Flu−PS: 3.18 mg g^−1^, 2.12 mg g^−1^, and 3.89 mg g^−1^, HA: 5.96 mg g^−1^, 4.28 mg g^−1^, and 6.36 mg g^−1^, and Flu−PS+HA: 4.17 mg g^−1^, 3.01 mg g^−1^, and 6.08 mg g^−1^, respectively). In a similar manner, the HA and Flu−PS+HA treatment groups showed lower ROS levels than the Flu−PS treatment groups. In addition, we discovered that the activity of the antioxidant enzymes superoxide dismutase and catalase was lower in the groups treated with HA and Flu−PS+HA than in the groups solely treated with Flu−PS. The results demonstrated that HA significantly lessens the toxicity caused by Flu−PS, while also promoting the germination and growth of *Solanum lycopersicum* seeds. The related decrease in toxic effects may be ascribed to the establishment of an eco-corona on the Flu−PS. We think that the use of eco-coronas is a technique for safeguarding plants against xenobiotics such as nanoplastics.

## 1. Introduction

The extensive use of plastic in industry and daily life represents a serious threat to the environment [1]. Nanoplastic contamination in the environment is caused by human activity, and plastic is widely used in agriculture (water supplying pipes, grow bags, plastic mulching, plastic pot, sewage water supply to crops, etc.), manufacturing, construction, and other industries. The consequence is that the world produced more than 311 Tg (million metric tons) of plastic in 2014, and it is growing at a pace of 20 Tg per year [2]. Due to their chemical inertness, plastics are often resistant to disintegration, and estimates for how long plastic garbage will remain in the environment range from decades to millennia [3]. In recent years, soil scientists have made progress in their knowledge of how nanoplastics affect terrestrial ecosystems, and innovative methods for measuring and identifying nanoplastics in the soil have been developed, tried, and evaluated [4]. The result of chemical changes to the polymer’s structure is degradation, which eventually results in a reduction in the mechanical strength of the plastic [5]. Less research has been carried out on the potential effects of plastic contamination in terrestrial environment [6]. Because deterioration has already started, it can proceed without additional UV exposure through temperature-dependent thermo-oxidative reactions, as long as oxygen is provided [7]. Due to their potential to have an impact on ecosystems, nanoplastics (NPs- < 1 µm) are of particular concern [8]. In some species, such as crops or plants that grow as single individuals or as a population but not in a community, nanoplastics have been observed to impact crop growth [6]. Nanoplastics have been demonstrated to have a greater impact on terrestrial and marine environments [9]. There is not much research that examines how crop development is impacted by high amounts of nanoplastics in agriculture and how this poses a risk to the food supply [4]. In the process of being deposited on the soil’s surface, nanoplastics are absorbed into the ground through a variety of processes, including biological activity [10]. From a scientific point of view, it is only rational to believe that plastic materials have a propensity to accumulate in soil [11], since there is a dearth of specific evidence about how plastic decomposes in soil [10]. One of the best indicators of agricultural production is the quantity of soil organic matter in the soil, which is a combination of plant, animal, and microbial wastes in various stages of decomposition [12]. New plant and animal wastes that break down in a matter of weeks to years make up the majority of the organic matter in active soil [13].

In addition to being investigated in the domains of soil chemistry, soil fertility, plant physiology, and environmental studies, humic compounds (humic acid and fulvic acid) account for 65–70% of the organic matter in soils [14]. Humic acid (HA) has a wide range of functions that can significantly benefit plant growth. Since it is in charge of several intricate chemical reactions in soil, HA is the component of soil organic matter that is the most prevalent (60%) and is recognized as an essential component of the terrestrial ecosystem [15]. Peats, lake sediments, shales, and brown coals are all examples of HA, which is a part of natural organic matter (NOM) and is common in both aquatic and terrestrial environments [16]. As a result of a chemical and microbiological breakdown, HA is produced [17]. Signal transduction, hormone metabolism, transcription, protein metabolism, transport, defense, and growth-related processes were all upregulated to a greater extent in the presence of HA, both in terms of the number of involved genes and fold change values. This study provides in-depth information on HA-dependent enhancement [18]. When organic biomolecules come into contact with nanoplastics, they compete for attachment to the hydrophobic surfaces of the nanoplastics, resulting in the production of an eco-corona (EC) [19]. Humic acid may adsorb nanoplastics and create a corona in *Daphnia magna* without precipitation, which causes certain alterations in the new-born *D. magna* in a fashion similar to microplastics, with the primary goal of reducing toxicity [20]. Extracellular polymeric substances (EPS), for instance, are a typical example of a biomolecule produced as a by-product of agricultural organisms’ metabolic processes [21]. Numerous studies have been conducted on the development of eco-coronas on plastic particles [22].

This research, which is the first of its type to examine the effects of fluorescent nanoplastics on tomato seeds, is based on a critical examination of earlier experiments with fluorescence nanoplastics. Only a few studies on the creation of eco-coronas over plastic particles have been documented in the literature, but it has not been determined whether or not this phenomenon can alter the harmful effects on tomato seeds and small plant growth. The goal of the current study was to determine if fluorescent nanoplastics had an impact on hydroponic seed-to-small plant development. This study’s main objective was to investigate the toxicity of nanoplastics such as Flu−PS, organic sources such as HA, and combinations of Flu−PS and HA on tomato seeds to plants (*Solanum lycopersicum*), and to determine whether the development of an eco-corona over the particles could help to lessen their toxicity. After interactions with Flu−PS, HA, and eco-coronated Flu−PS+HA with *Solanum lycopersicum*, seed germination and root and shoot length measurements were recorded microscopical pictures (of seeds and plants) were taken, chlorophyll estimation was carried out and reactive oxygen species formation evaluation and antioxidant enzyme activity evaluation were performed.

## 2. Results

### 2.1. Characterization Study

#### 2.1.1. Particle Size

Flu−PS particles had a diameter of 235.5 nm, and the mixture of both Flu−PS+HA complexes had a diameter of 325.9 nm, and the difference between Flu−PS and Flu−PS+HA was 90.4, while the size of the Flu−PS particle increased as it combined with HA. Flu- PS and Flu−PS+HA had z-potential values of 89.7 mV and −96.6 mV, respectively (Table 1).

#### 2.1.2. Transmission Electron Microscope (TEM)

**Figure 1:**
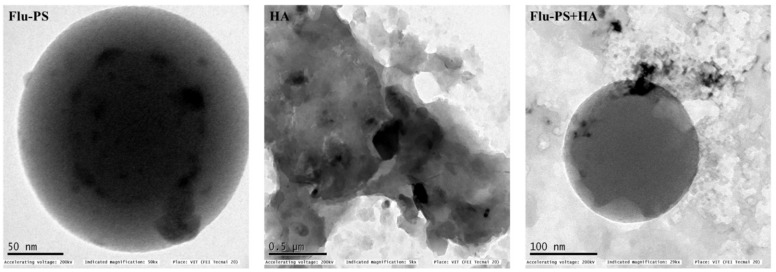
Transmission electron microscopy (TEM) image of Flu−PS, HA and Flu−PS+HA dispersed in deionized water.

The shape of the Flu−PS particles dispersed in deionized water was revealed to be spherical by structural characterization of the samples using TEM, as indicated by the manufacturer. However, humic acid dispersed in deionized water was observed in irregular shapes, as expected. Furthermore, Flu−PS+HA dispersed in deionized water demonstrated a clear formation of eco-coronas, and humic acid was adsorbed on the surface of PS (Figure 1).

#### 2.1.3. Fourier Transform Infrared (FTIR)

**Figure 2:**
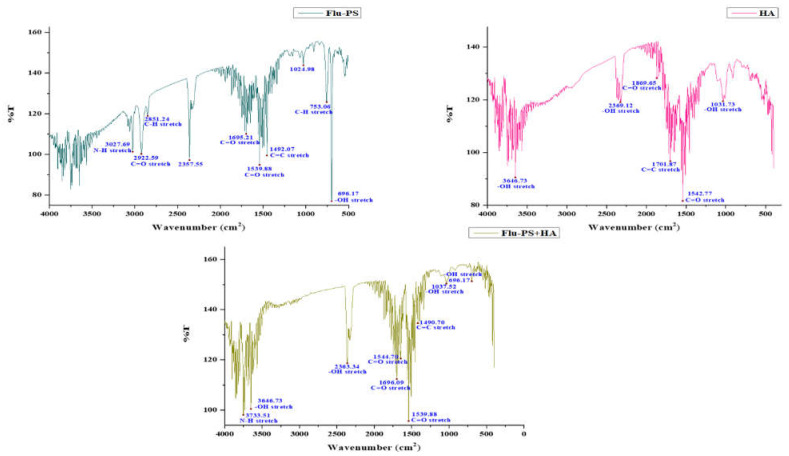
Fourier transform infrared spectroscopy image of Flu−PS, HA, and Flu−PS+HA.

FTIR spectra were used to locate the active functional groups engaged in the binding. Significant IR bands were observed in the Flu−PS suspension at 3027.69 cm^−1^, 2922.59 cm^−1^, 2851.24 cm^−1^, 2357.55 cm^−1^, 1695.21 cm^−1^, 1539.88 cm^−1^, 1492.07 cm^−1^, 1024.98 cm^−1^, 753.06 cm^−1^ and 696.17 cm^−1^. At 1492.07 cm^−1^, the peak created corresponds to C=C stretching vibrations in polystyrene. Strong IR bands were identified in the HA suspension at 3646.73 cm^−1^, 2369.12 cm^−1^, 1896.65 cm^−1^, 1701.87 cm^−1^, 1542.77 cm^−1^, and 1031.73 cm^−1^. The peak at 1542.77 cm^−1^ reflected C=O stretching vibrations in humic acid. Strong IR bands were found in the Flu−PS+HA suspension at 3733.51 cm^−1^, 3646.73 cm^−1^, 2363.34 cm^−1^, 1696.09 cm^−1^, 1544.70 cm^−1^, 1539.88 cm^−1^, 1490.70 cm^−1^, 1037.52 cm^−1^ and 696.17 cm^−1^. The peak at 1490.70 cm^−1^ and 1544.70 cm^−1^ represented C=C and C=O stretching vibrations in both combinations of Flu−PS+HA (Figure 2).

#### 2.1.4. Three-Dimensional Fluorescence Spectroscopy Analysis

**Figure 3:**
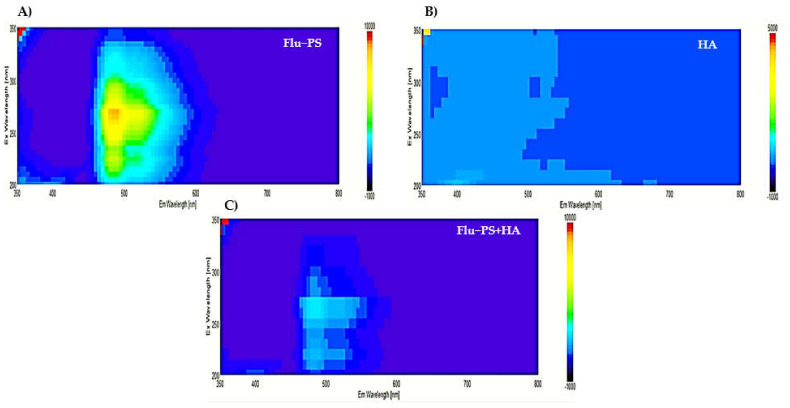
The3D Fluorescence spectroscopy observation pattern images of (**A**) Flu−PS, (**B**) HA, and (**C**) Flu−PS+HA.

In the 3D fluorescence spectra of Flu−PS, HA, and Flu−PS+HA, the following peaks were identified: Flu−PS from 5 mg/L was observed to peak at the Ex/Em wavelength of 270–280/490–500 nm, represented by the fluorescence of Flu−PS. For HA, no peak was observed in the organic source. For Flu−PS+HA, there was an obvious fluorescence peak that was observed, mainly resulting from the decrease in PS activity at the Ex/Em wavelength of 275–285/495–500 nm. Humic acid affected the fluorescence of nanoplastics, as shown in Figure 3 for Flu−PS+HA.

#### 2.1.5. X-ray Diffraction (XRD)

**Figure 4:**
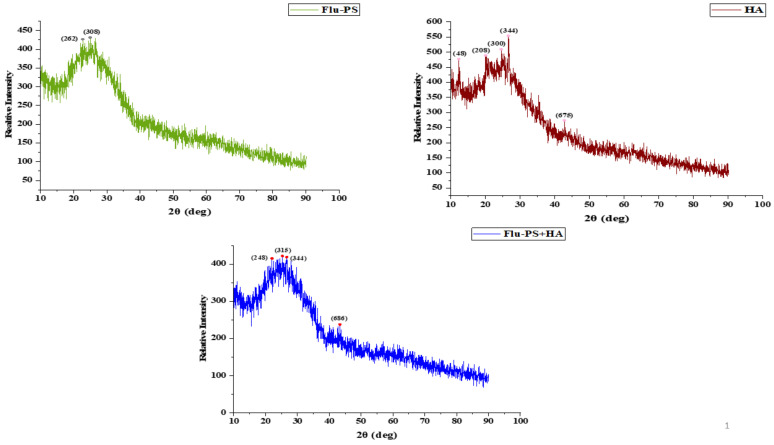
XRD observation patterns: Flu−PS, HA, and Flu−PS+HA.

Flu−PS, HA, and Flu−PS+HA XRD patterns were created (Figure 4). The peaks demonstrated by XRD were angled (2) at 22.70° and 24.93°, corresponding to the Flu−PS planes (262) and (308), respectively. The peaks at angles (2) were 24.55°, 26.59°, and 42.79°, which correspond to the HA planes (300), (344), and (675). The peaks located at angles (2) 22.01°, 25.28°, and 43.33° correspond to the Flu−PS+HA complex planes (248), (315), and (686), respectively.

### 2.2. Effect of Flu−PS, HA, and Flu−PS+HA on Solanum lycopersicum (Seeds)

#### 2.2.1. Seed Imaging by Fluorescence Optical Microscopy

**Figure 5:**
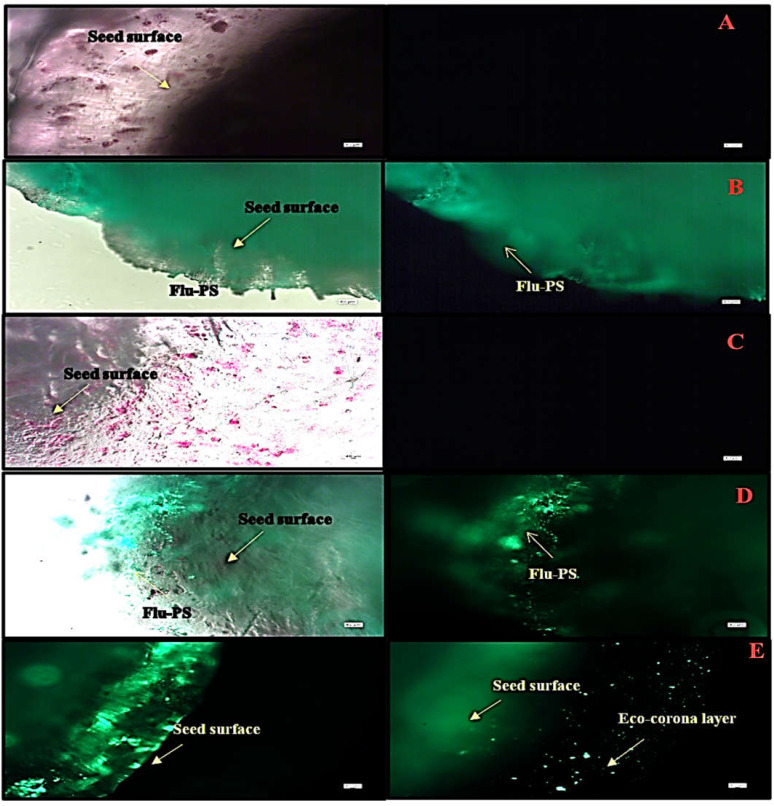
Fluorescence microscopic image of (**A**) the control, (**B**) Flu−PS, (**C**) HA, (**D**) Flu−PS+HA, and (**E**) Flu−PS+HA on *Solanum lycopersicum* seeds. (**B**) Flu−PS interacted with seeds after 24 h; the microscopic image shows a lesser number of healthy cells on the surface of the seed. (**A**) The control and (**C**) HA microscopic image shows a higher number of healthy cells on the surface of the seed and (**D**) the Flu−PS+HA microscopic image shows evidence of a higher number of healthy cells; on the surface of the seed, an eco-corona layer was observed.

Under a fluorescence optical microscope, the seeds interacted with three different groups, as well as a control group. Before germination, nanoplastic accumulates on the seed surface, particularly in the surface pores, as evidenced by the strong green fluorescence, indicating the accumulation of multiple nanoplastics. The seeds, as shown in Figure 5A–E, interacted with Flu−PS, HA, and Flu−PS+HA treatments along with control. When the seeds were treated with Flu−PS+HA for 24 h, the surface of the seeds formed an eco-corona.

#### 2.2.2. Seed Germination

**Figure 6:**
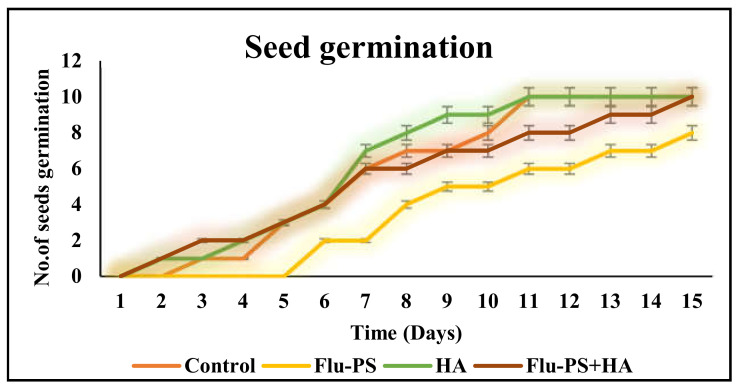
The seed germination rate for *Solanum lycopersicum* is shown in this line graph (control, Flu−PS, HA, and Flu−PS+HA).

Flu−PS, HA, and their combinations all had a significant impact on seed germination. On day 8, the seeds in the control group began to germinate. Flu−PS-treated seeds, on the other hand, germinated after 15 days. Flu−PS exposure delayed seed germination by seven days. HA-treated seeds germinated on day 8, as was the case for the control group. Surprisingly, seeds treated with the Flu−PS+HA combination germinated significantly earlier on day 7. Seed germination rates for the Flu−PS-, HA-, and Flu−PS+HA-treated groups were 63%, 89%, and 98%, respectively, with the control showing 100% germination. This suggests that Flu−PS and HA should be combined to promote seed germination (Figure 6). However, seed germination was greater for eco-coronated Flu−PS-treated seeds compared to Flu−PS. Although HA had a positive impact on root size and shoot length, Flu−PS applications did not affect these parameters.

### 2.3. Effect of PS, HA, and PS+HA on Solanum lycopersicum (Plants)

#### 2.3.1. Shoot and Root Length

**Figure 7:**
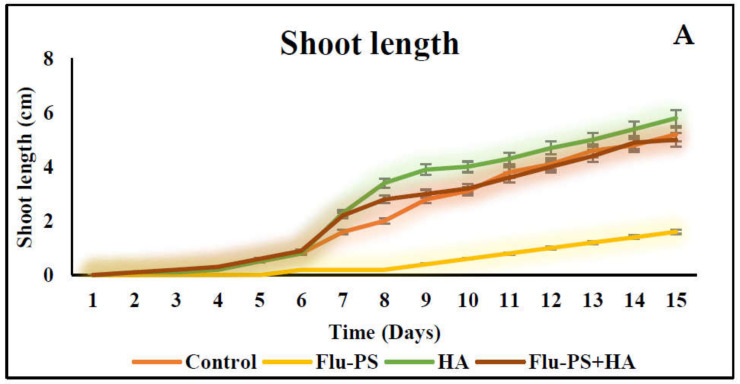

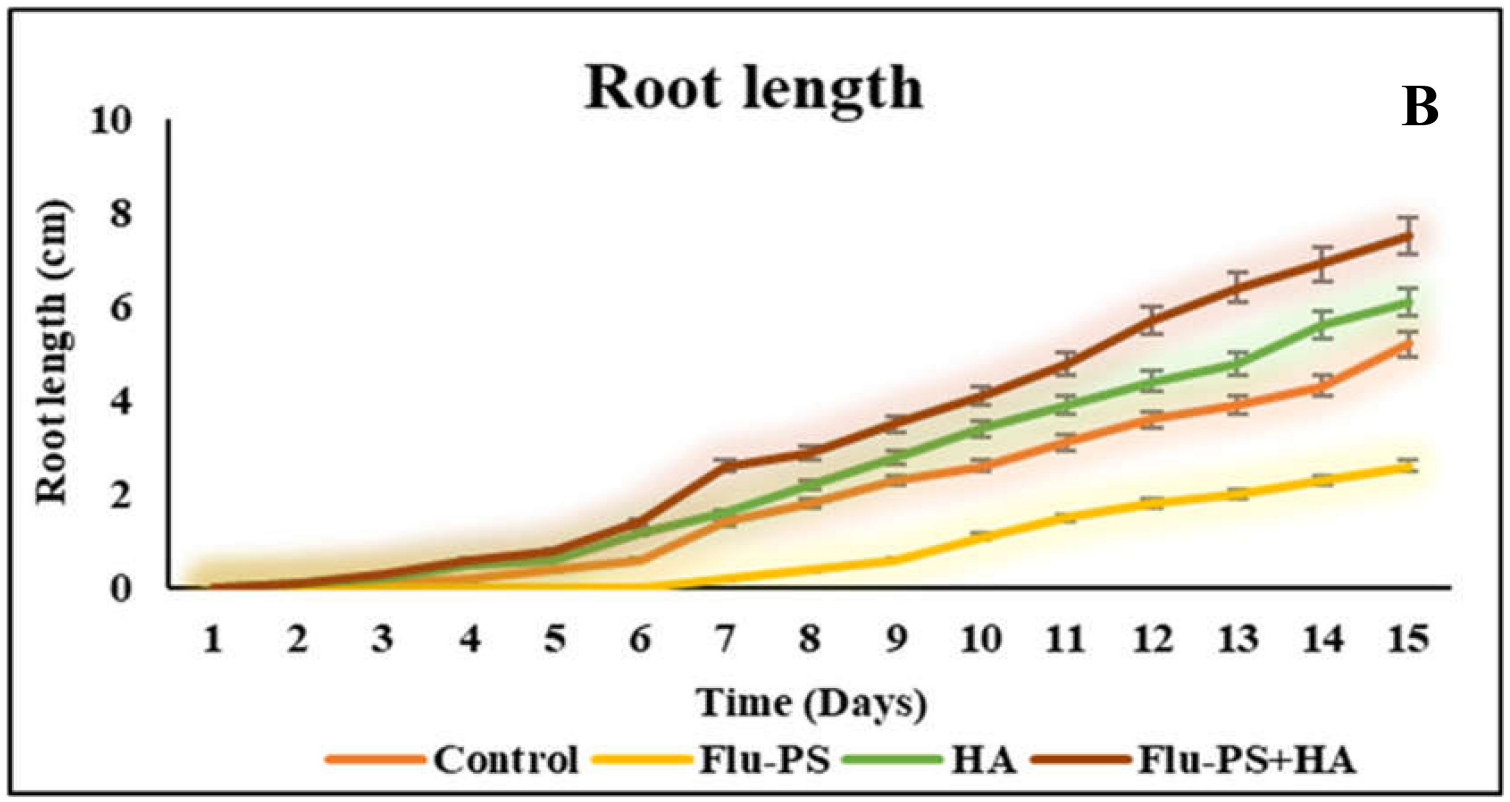
Measurements of the shoot and root lengths are shown in this line graph. (**A**) represents shoot length and (**B**) represents root length in *Solanum lycopersicum* (Control, Flu−PS, HA and Flu−PS+HA).

**Figure 8:**
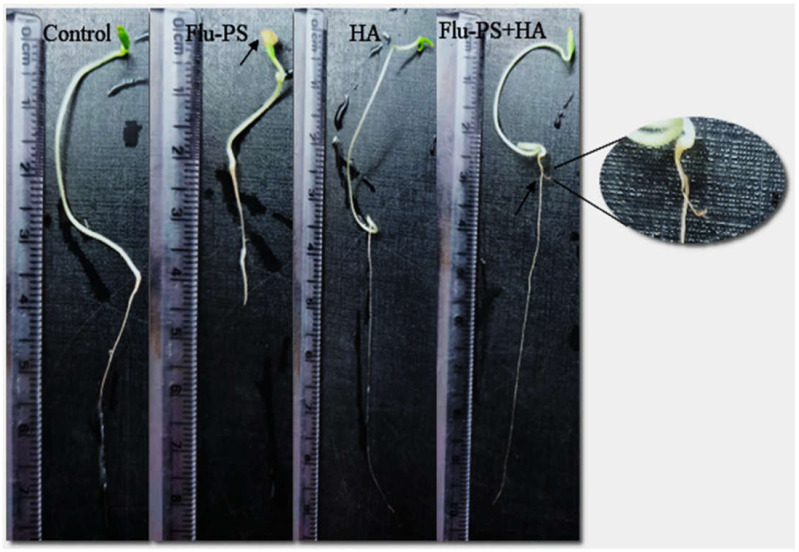
Effect of Flu−PS, HA, and Flu−PS+HA treatments, as well as control (untreated), on *Solanum lycopersicum* plant over 8 days. Flu−PS shows on the ninth day that the leaf has not been penetrated, while the control and HA showed a full-grown plant. Flu−PS+HA also showed a full-grown plant, followed by secondary root penetration.

The average shoot and root lengths of the plants in the control group on the fifteen days were 5.2 cm and 5.2 cm, respectively. The Flu−PS-treated group, on the other hand, had shoot and root lengths of 1.6 cm and 2.6 cm, respectively. The shoot and root lengths in the HA-treated set were 5.8 cm and 6.1 cm, respectively. The shoot and root lengths of the Flu−PS+HA complex, on the other hand, were 5 cm and 7.5 cm, respectively (Figure 7A,B). The HA- and Flu−PS+HA-treated groups had the longest shoot and root lengths. Figure 8 shows pictures of the Flu−PS, HA, and Flu−PS+HA-treated plants as well as the control.

#### 2.3.2. Plant Imaging by Optical Microscopy

**Figure 9:**
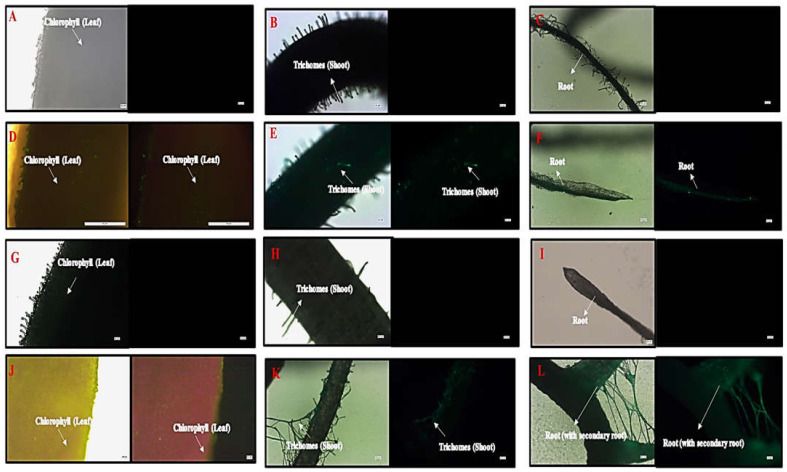
Microscopic images of control plant, ((**A**). leaf, (**B**). shoot, (**C**). root), Flu−PS-interacted plant ((**D**). leaf (with light, and under fluorescence light), (**E**). shoot (with light, and under fluorescence light), (**F**). root (with light, and under fluorescence light)), HA-interacted plant ((**G**). leaf, (**H**). shoot, (**I**). root) and Flu−PS+HA-interacted plant ((**J**). leaf (with light, and under fluorescence light), (**K**). shoot (with light, and under fluorescence light), (**L**). root (with light, and under fluorescence light)).

A microscopic image revealed hair-like structures known as trichomes in the shoots of the control, HA, and in the Flu−PS+HA accumulation of green fluorescence nanoplastics on the surface, along with healthy cells that were present on the surface-treated groups. On the other hand, only a few trichomes were found on the shoots along with the surface, as shown by the strong green fluorescence, indicating the accumulation of multiple nanoplastics in Flu−PS-treated plants. Furthermore, when the features of the root were examined, well-grown root hairs were observed on the surface of the root in both the control and HA groups. However, the Flu−PS-treated groups had a significantly lower number of root hairs, indicating that Flu−PS had negatively impacted the root system, impairing the development and growth of root hairs, which are essential parts of the plant for the uptake of water and nutrients from the soil. This, in turn, may affect the shoot’s growth and elongation. This reinforces the impact of Flu−PS on the development of the entire root system. Surprisingly, treatment with humic acid alleviated Flu−PS’s negative effect on the development and growth of root hairs. The development of root hairs in Flu−PS+HA-treated groups was as normal as in the control group, with a comparatively high number of root hairs, indicating the protective role of humic acid after it formed an eco-corona around Flu−PS. Furthermore, the Flu−PS+HA group had secondary roots. Humic acid, which formed an eco-corona around Flu−PS, significantly reduced Flu−PS’s negative effect on root hair development, and thus plant root health. Secondary roots were found in the Flu−PS+HA complex as well. The presence of root hairs was significantly reduced in the Flu−PS-treated plant (Figure 9).

#### 2.3.3. Photosynthetic Pigment Estimation

Chlorophyll (Chl) a, b, and total chlorophyll (a + b) were measured in leaves pierced by Flu−PS-, HA-, and Flu−PS+HA-treated seeds. Chlorophyll levels in the control plants and plants treated with HA differed significantly. When the chlorophyll concentration of all the treated plants was compared, those treated with pure Flu−PS had reduced chlorophyll content.

**Figure 10:**
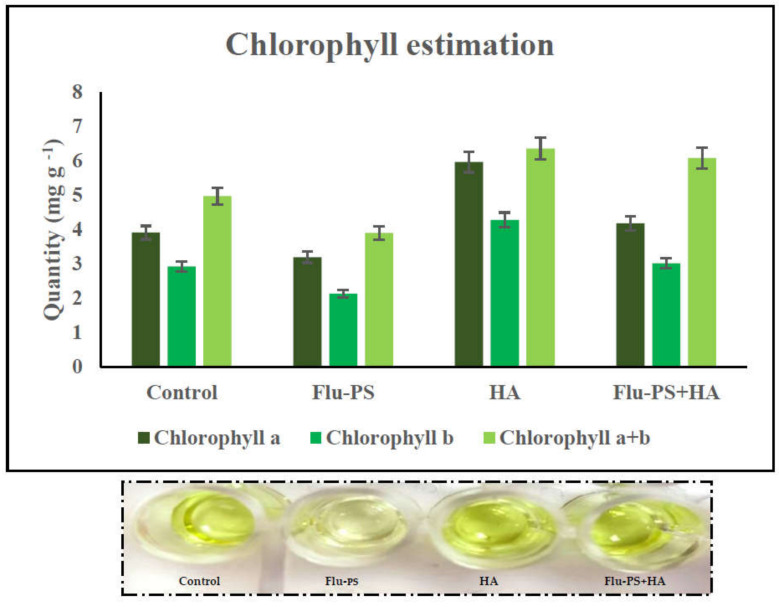
Chlorophyll estimation: effects of the control, Flu−PS, HA and Flu−PS+HA on *Solanum lycopersicum*.

The analysis revealed that the Chl a range in the control leaf samples was 3.90 mg g^−1^, the Chl b range was 2.92 mg g^−1^, and the total Chl (a + b) range was 4.97 mg g^−1^. The Chl a range in Flu−PS-treated leaf samples was 3.18 mg g^−1^, the Chl b range was 2.12 mg g^−1^, and the overall Chl (a + b) range was 3.89 mg g^−1^. The Chl a range in HA-treated leaf samples was 5.96 mg g^−1^, the Chl b range was 4.28 mg g^−1^, and the overall Chl (a + b) range was 6.36 mg g^−1^. As indicated in the figure, the Chl a range in eco-coronated Flu−PS+HA-treated leaf samples was 4.17 mg g^−1^, the Chl b range was 3.01 mg g^−1^, and the overall Chl (a + b) range was 6.08 mg g^−1^ (Figure 10).

### 2.4. Oxidative Stress Analysis

#### 2.4.1. ROS Production

**Figure 11:**
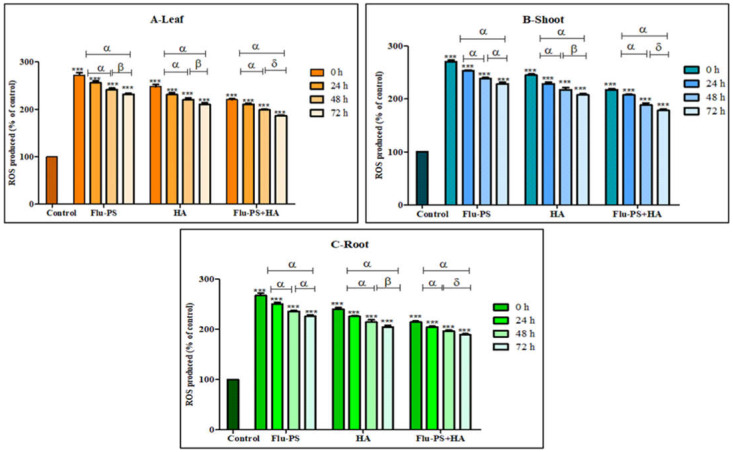
Intracellular reactive oxygen species (ROS) values of Flu−PS-, HA- and Flu−PS+HA-treated ((**A**). Leaf, (**B**). Shoot, (**C**). Root) *Solanum lycopersicum* samples. Note: ‘ *** ’ indicates the percentage difference with respect to the control; ‘α, β, δ’ indicates a significant difference (α = *p* < 0.001, β = *p* > 0.001, δ = *p* > 0.05).

The total ROS for Flu−PS-, HA-, and Flu−PS+HA-treated plants (leaf, shoot, and root) were studied using DCFH-DA fluorescent dye (Figure 11). ROS production in all the samples (Flu−PS, HA, and Flu−PS+HA) significantly increased when compared to the control (*p* < 0.001). When a comparison was made between the time points, ROS production in PS-treated samples at 0 h to 24 h, and 0 h to 72 h (leaf, shoots, and roots) was significantly higher (*p* < 0.001), when compared to samples at 48 h to 72 h (leaf) (*p* > 0.001) and 48 h to 72 h (shoot and root) (*p* > 0.001). In HA-treated samples, ROS production decreased as follows: from 0 h to 24 h, and 0 h to 72 h (leaf, shoots, and roots), it was significantly higher (*p* < 0.001) when compared to samples at 48 h and 72 h (leaf, shoots, and roots) (*p* > 0.001). The difference between the time points demonstrated a highly significant decrease in overall ROS generation in 48 h and 72 h samples, when compared to 24 h samples (leaf, shoot, and root).

Furthermore, the eco-coronation of Flu−PS decreased the ROS production at all the time points when compared to Flu−PS-treated samples. In eco-coronated Flu−PS, the samples showed a decrease in the ROS generation at 0 h to 24 h and 0 h to 72 h (leaf, shoot, roots), which was significant (*p* < 0.001). At 48 h 72 h, decreasing significant changes were observed (*p* > 0.05), but in the case of shoots at 48 h and 72 h, no significant were changes observed (*p* > 0.05). However, in the graph, it is quite evident that the eco-coronated treated plant results showed decreased total ROS (*p* < 0.001), with increasing time of treatment.

#### 2.4.2. Effects on Superoxide Dismutase Activity

**Figure 12:**
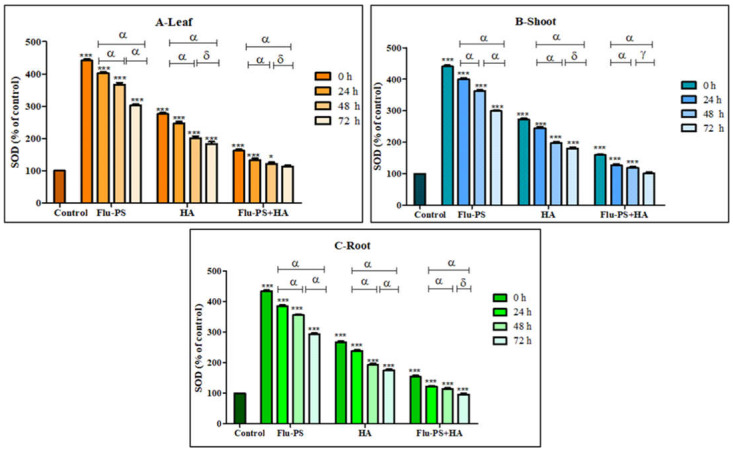
Percentage of superoxide dismutase (SOD) values of Flu−PS-, HA- and Flu−PS+HA-treated ((**A**). Leaf, (**B**). Shoot, (**C**). Root) *Solanum lycopersicum* samples. Note: ‘ * *** ’ indicates the percentage difference with respect to the control; ‘α, δ, γ’ indicates a significant difference (α = *p* < 0.001, δ = *p* > 0.05, γ = *p* < 0.05).

All the treated (Flu−PS, HA, and Flu−PS+HA) plants (leaf, shoots, and roots) showed a significant increase (*p* < 0.001) in the SOD activity, when compared with the control samples (Figure 12). Flu−PS-treated samples (leaf, shoots, and roots) showed a highly significant increase (*p* < 0.001) when compared to the SOD activity of the control samples from 0 h to 24 h, 24 h to 48 h, and 0 h to 72 h. In HA-treated samples, ROS production decreased as follows: from 0 h to 24 h, and 0 h to 72 h (leaf, shoots, and roots), ROS production was significantly higher (*p* < 0.001) when compared to samples at 48 h and 72 h (leaf and shoot) (*p* > 0.05) and 48 h and 72 h (root) (*p* < 0.001). The difference between the time points demonstrated a highly significant decrease in overall ROS generation in the 48 h and 72 h samples, when compared to the samples from 0 h to 24 h and 0 h to 72 h, which were reported to be significant (leaf, shoot, and root).

Furthermore, eco-coronation of Flu−PS decreased the SOD activity at all the time points when compared to the Flu−PS-treated samples. In eco-coronated Flu−PS, the samples showed a decrease in the SOD activity at 0 h and 24 h (leaf, shoots, and roots), which is significant (*p* ≤ 0.001). For the leaves and roots, at 48 h to 72 h, no significant changes were observed (*p* > 0.05). This was also the case in the shoots, at 48 h to 72 h, no significant change was observed (*p* < 0.05). However, in the case of eco-coronated Flu−PS-treated plants, the results showed positive effects of SOD activity with increasing time of treatment.

#### 2.4.3. Effects on Catalase Activity

**Figure 13:**
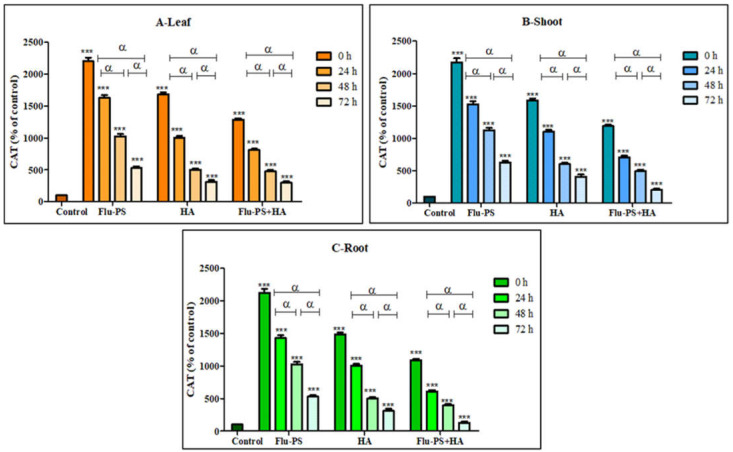
Percentage of Catalase values of Flu−PS, HA & Flu−PS+HA treated ((**A**). Leaf, (**B**). Shoot, (**C**). Root) *Solanum lycopersicum* samples. Note: ‘ *** ’ indicates the percentage difference concerning the control, and ‘α’ indicates a significant difference (α = *p* < 0.001).

Catalase activity in the sample (Flu−PS, HA, and Flu- PS+HA)-treated plants (leaf, shoots, and roots) is illustrated in Figure 13. Flu−PS-treated plants (leaf, shoots, and roots) showed a significant increase (*p* ≤ 0.001) in catalase production with the control samples. Furthermore, it is observed that Flu−PS-treated plant (leaf, shoots, and roots) samples had higher significance when compared to their respective HA and eco-coronated Flu−PS-treated samples (*p* ≤ 0.001).

## 3. Discussion

Once in agroecosystems, nanoplastics remain, accumulate, and eventually reach levels that affect biodiversity and ecosystem function. However, the potential biological effects of nanoplastics on plant development, as well as the mechanisms underlying nanoplastic actions, are largely unknown. The size of eco-coronated Flu−PS nanoplastics increased gradually. These findings imply that HA-formed eco-coronated Flu−PS has a higher affinity for plant cell binding. The surface charge of nanoplastics in the agricultural environment regulates their stability and aggregation behavior, which determines their colloid behavior. Because seed germination is such an important stage in the plant’s life cycle, it has been widely used as an index to analyze the phytotoxicity of hazardous chemicals [23]. The dynamic light scattering and zeta potential (Table 1) results showed that the Flu−PS and Flu−PS+HA dispersed in deionized water formed a stable dispersion of particles, with sizes of approximately 235.5 nm and 325.9 nm, respectively. The overall charge on Flu−PS and Flu−PS+HA, regardless of surface functionalization, could be caused by the presence of Flu−PS (carrying a positive charge) and eco-corona-formed Flu−PS (carrying a negative charge) [24]. According to these findings, negatively charged HA has a higher binding affinity on the seed surface than positively charged Flu−PS. In another study by Giri et al., positively charged PS was found to have a higher toxicity rate toward the yeast cells of *Saccharomyces cerevisiae* compared to negatively charged PS [25]. The eco-corona formation is revealed by the TEM image results for Flu−PS (Figure 1), HA (Figure 1), and Flu−PS+HA (Figure 1). Another study by Miyazaki et al. obtained consistent TEM images of eco-coronas on the surface of the PS with nanoplastics in the marine diatom *Phaeodactylum tricornutum* [26]. It is assumed that infrared spectroscopy is a qualitative tool for determining the presence of functional groups in Flu−PS, HA, and Flu−PS+HA (Figure 2). C=C groups were discovered to be detectable by PS bands at 3000–3100 cm^−1^ and 1668 cm^−1^ [27,28]. The bands at 1542.77 cm^−1^ and 1730 cm^−1^ demonstrated the presence of C=O groups in HA [28]. Figure 3 shows the Flu−PS 3D fluorescence spectroscopy results at 270–280/490–500 nm [29]. A HA fluorescence peak was not observed, but Flu−PS+HA at 275–285/495–500 nm demonstrated reduced nanoplastic PS fluorescence. After 24 h, the interaction of Flu−PS+HA suspension with tomato seeds resulted in eco-corona formation on the seeds’ surface. The Flu−PS XRD peak can be observed at 22.70° in Figure 4, which was similar to another study by Könemann et al. that obtained a peak at 18.8° [30]. The HA XRD peak was found to be 42.79°, which was similar to another study by Niculăescu et al. that found the peak to be 47.45° [31].

Microscopical images (Figure 5) revealed that very few healthy cells are present on the surface of the Flu−PS seed, whereas a greater number of healthy cells are present on the surface of the HA and combination of Flu−PS+HA seeds. The toxic effects were reduced by the interaction of humic acid with Flu−PS due to the surface eco-corona layer. These microscopical images are the first visible result of eco-corona development on the seeds’ surface. Importantly, humic acid promotes nutrient cycling, which promotes growth [32]. Another study by Giri et al. discovered that when *Allium cepa* roots were exposed to eco-coronated PS, the particle size increased [33]. The seed germination rate graphs (Figure 6) indicated that in comparison to Flu−PS and HA, in Flu−PS+HA, the seed germination rate is higher, as also observed in the images (Figure 7). The (A) shoot length and (B) root length results indicated that the Flu−PS+HA- and HA-treated shoots and roots were longer than those for Flu−PS. Figure 8 showed, for the Flu−PS plant, that the leaves had not yet been penetrated, by which time the HA and Flu−PS+HA plants were showing matured small leaves. In Flu−PS+HA plants, secondary root formation was also observed. Microscopical images revealed that PS-treated plants had fewer trichomes in the shoot, root hair in the root, and leaf hair in the leaf than HA and PS+HA-treated plants, as represented by (Figure 9). As a result, the effect of humic acid on the plant was investigated, and it was discovered that it helped to improve soybean yield in the field [34]. Another researcher also discovered that humic acid improves plant growth in maize [35]. In comparison to control plants, HA- and Flu−PS+HA-treated plants had a greater number of trichomes in the shoot, root hair in the root, and leaf hair in the leaf. Secondary root formation was also observed in Flu−PS+HA-treated plants. Because of eco-corona formation, the root and shoot lengths were found to be equal in HA- and Flu−PS+HA-treated plants compared to others. In terms of chlorophyll estimation, Chl a, Chl b, and both Chl a + b were evaluated. The contents of Chl results from this study suggest that a, b, and a + b were higher in HA-treated and eco-coronated Flu−PS-treated plants, but significantly lower in Flu−PS-treated plants (Figure 10). A similar result was found in a study by Lian et al. [36], where the PS reduced the chlorophyll in lettuce (*Lactuca sativa* L.). Humic acid treatment increased chlorophyll pigment production in common bean plants, yielding a similar result (*Phaseolus vulgaris* L.) [37]. HA is likely to be highly adsorbed by plastic particles due to the presence of carboxylic and phenolic groups in its structure, as well as an abundance of functional groups, such as methoxyl, hydroxyls, ketones, and quinines [38]. Similar results were observed in Lemna minor, where HA reduced AgNP toxicity [39]. To fully understand the effects of PS, additional research on the effects of PS on total ROS production in plant systems is required. Reactive oxygen species appear to be the primary mediator of oxidative stress [40]. ROS are produced in the essential cellular process in photosynthetic systems, such as in plants [41]. Antioxidant enzymes are indirect indicators of cellular oxidative stress, and superoxide dismutase and catalase are two widely used enzymes. Furthermore, PS-induced membrane damage is linked to their production [42]. Recent research with *Scenedesmus obliquus* discovered a comparable reduction in ROS generation by PS when fulvic and humic acids were present (HA and FA) [43]. PS caused extensive oxidative damage in *Solanum lycopersicum* in terms of ROS, and HA reduced its toxicity. In addition, HA reduced the toxic effects of AgNPs in *Lemna minor* plants [39]. In this experiment, ROS activity was higher in the Flu−PS-treated samples (leaf, shoot, and root) than in the untreated (control) samples, and a lower quantity of ROS was observed in eco-coronated Flu−PS (leaf, shoot, and root) (Figure 11). A similar reduction in ROS production by PS in the presence of HA was observed in a recent study with *Scenedesmus obliquus* [43]. Furthermore, when tomato leaves, shoots, and roots were exposed to Flu−PS, CAT activity increased, while SOD activity decreased dramatically. When compared to the control, the eco-coronated form of Flu−PS caused a lower increase in both ROS levels and a lower increase in CAT activity and SOD activity. As a result, the greater levels of eco-coronation in Flu−PS resulted in a significant decrease in SOD activity (Figure 12), as well as a decrease in CAT activity and SOD activity (Figure 13). The same trend of decreased CAT activity in *Synechococcus* sp. after treatment with fulvic acid (a variant of NOM)-coated iron nanoparticles has previously been reported [44]. The same trend of decreased SOD activity in *Allium cepa* root has also been reported in eco-coronated PS [33].

In the current study, increased oxidative stress, as mentioned in the preceding sections, increased the activities of the antioxidant enzyme catalase. The enzyme activity was significantly reduced when the coronated Flu−PS interacted with the seed cells. Because of their nano size, Flu−PS could easily enter seed cells, generating ROS and reducing plant physiology. As the size of Flu−PS particles increased with eco-corona development, the nanoplastic became impermeable to the seeds, increasing the number of healthy cells, seed germination, ROS production, and antioxidant enzyme activity.

## 4. Materials and Methods

### 4.1. Seed Collection

VIT School of Agriculture and Advanced Learning (VAIAL), VIT University, Vellore, Tamil Nadu, India provided the *Solanum Lycopersicum* seeds utilized in this research. For 10 to 15 min, the seeds were steeped in distilled water. The seeds were chosen using the float/sink test. Seeds that floated to the top were rejected, while those that sunk to the bottom were chosen for future research. Selected seeds were washed with distilled water, before being immersed in 80% ethanol for 2 min at room temperature. After rinsing with ethanol, they were rinsed with deionized water 5 times and immersed with 60% ethanol, and then rinsed with deionized water. These surface sterilizations were carried out to prevent fungal infection. All of the tests were carried out in triplicate.

### 4.2. Experimental Design

Polystyrene green fluorescently labeled nanoplastics (Flu−PS-200 nm size) were obtained from Corpuscular Inc., Philipstown, NY, USA, and organic humic acid (HA) from Sigma Aldrich, India. The experimental Flu−PS concentration was 5 mg/L, prepared from a stock solution of 25,000 mg/L, and the humic acid (HA) concentration was 5 mg/L. Flu−PS and HA were mixed in a 1:1 ratio to study their interaction. In a sterile glass Petri dish (diameter: 100 × 15 mm), 6 sterile *Solanum lycopersicum* seeds were placed on Whatman filter paper (125 mm). Using the hydroponic method, a treatment solution that contained polystyrene with or without humic acid was added at the top and bottom of the filter paper (each in a separate plate in triplicates). The plates were kept at room temperature with a relative humidity of 50% with the lids closed. The experimental setup remained untouched until the seeds germinated. The experiment lasted one to two weeks, with regular re-supply of treatment solution (by adding it superficially) twice a day. The control was provided by deionized water.

### 4.3. Characterization Study

#### 4.3.1. Particle Size Determination

The dynamic light scattering (DLS) method was used to estimate the particle size of the Flu−PS and Flu−PS+HA. A diode-pumped frequency double laser at 532 nm, (10 mW) with light scattering at an angle of 173°, was used to estimate the particle size of the Flu−PS and Flu−PS+HA dispersed in deionized water [45]. The data were collected and analyzed using the manufacturer’s recommendations (SZ-100 software). Similarly, for the zeta potential (Z-potential) of the Flu−PS and Flu−PS+HA, the electrophoretic mobility (cm^2^/V-s) of the particles was converted to zeta potential (milli-volts-mV) and assessed using the provided SZ-100 software and both DLS and Z-potential were analyzed using a nanoparticle analyzer (HORIBA, Kyoto, Japan).

#### 4.3.2. Transmission Electron Microscopy

Transmission electron microscopy was used to determine the size and shape of the Flu−PS, HA, and Flu−PS+HA. Dispersed in deionized water samples [46], Flu−PS, HA, and Flu−PS+HA were placed on a copper grid with a carbon coating, and one section of the suspension was imaged using transmission electron microscopy (TEM; JEOL 1010, JEOL Ltd., Tokyo, Japan-HRTEM).

#### 4.3.3. FTIR-Fourier Transform Infrared Spectroscopy Analysis

The samples (Flu−PS, HA, and Flu−PS+HA) dispersed in deionized water were examined [45] using an (JASCO FTIR-6800, Tokyo, Japan) at a specific resolution (scan) of 4 cm^−1^, for the validation of n-characteristic functional groups. The analysis was carried out in the spectral range of 4000 to 400 cm^−1^.

#### 4.3.4. Three-Dimensional Fluorescence Spectroscopy Analysis

A spectrofluorometer was used to detect three-dimensional fluorescence (3D Fluorescence) at room temperature (JASCO FP-8300, Tokyo, Japan). Flu−PS, HA, and Flu−PS+HA dispersed in deionized water were subjected to spectrum analysis [45]. The excitation (λ_ex_) wavelength ranged from 200 to 350 nm, whereas the emission (λ_em_) wavelength ranged from 350 to 800 nm. The scanning speed was 1000 nm/min. The spectrum of deionized water served as the control.

#### 4.3.5. X-ray Diffraction (XRD) Analysis

To evaluate the powder structure of the samples, X-ray diffraction analysis was performed. The samples (Flu−PS, HA, and Flu−PS+HA) dispersed in deionized water [45] were evaluated using a(BRUKER D8- Advance P-XRD, Karlsruhe, Germany) source 2.2-kilowatt Cu-anode ceramics tube. The instrument was built with a Lynx Optic Detection System (silicon strip detection technique) and a reflectance detector (for low-angle detection).

### 4.4. Seeds and Plant Growth

Seeds of *Solanum lycopersicum* grown hydroponically in Petri dishes were evaluated for various growth parameters, such as seed germination, root length, and shoot length. A few drops of PBS were added to the samples on a glass slide covered with a coverslip to keep them moist [47]. The surface of the seeds, root, and shoot was examined under a 40× magnification optical microscope (LEICA DM-2500, Wetzlar, Germany).

### 4.5. Plant Physiology

#### Photosynthetic Pigment Measurement

Chlorophyll absorbs light during photosynthesis; there are two types of chlorophyll, a and b. Chlorophyll a donates electrons, whereas chlorophyll b allows organisms to absorb more blue light for photosynthesis. Around 0.2 g of leaf germinated from tomato seeds was finely cut and separated in sterile glass tubes. About 2.5 mL of 80% acetone and 0.03125 g of softly crushed magnesium carbonate powder were added to each tube. Using a mortar and pestle, the leaf was gently ground. The samples were then incubated at 4 °C for 3 h. Following incubation, all samples were centrifuged at 2500 rpm for 5 min.

The aqueous phase (supernatant) was transferred to a sterile glass tube, which was then filled to 2 mL with 80% acetone and used to quantify chlorophyll. The absorbance of the solutions was measured using a UV–visible spectrophotometer (Hitachi, U-2910, Japan) at 645 nm and 663 nm (max for chlorophyll a and b, respectively), with an acetone solution of 80% as a blank. Averaging the results of three measurements yielded an estimate of the chlorophyll concentration. The quantities of chlorophyll a, b, and a + b were calculated using the equations below [48].

(1) $$\mathrm{chlorophyll}\;a\;(\mathrm{mg}/g\;\mathrm{tissue})=\frac{12.7\left(A663\right)-2.695\left(A645\right)\times \;V}{1000\times W}$$

(2) $$\mathrm{chlorophyll}\;b\;(\mathrm{mg}/g\;\mathrm{tissue})=\frac{22.9\left(A645\right)-4.68\left(A663\right)\times \;V}{1000\times W}$$

(3) $$\mathrm{total}\;\mathrm{chlorophyll}\;a+b\;(\mathrm{mg}/g\;\mathrm{tissue})=\frac{20.2\left(A645\right)+8.02\left(A663\right)\times \;V}{1000\times W}$$

where A is the absorbance at a specific wavelength, V is the final volume of chlorophyll extract in 80% acetone, and W the fresh weight of the tissue extracted.

### 4.6. Oxidative Stress Analysis

#### 4.6.1. Sample Preparation for Oxidative Stress Analysis

All (treated) and control (untreated) leaf, root, and shoot samples were finely cut and ground using a mortar and pestle. To homogenize the samples, 2 mL of 0.5 M phosphate buffer was added to each sample. Plant samples were collected at regular intervals of 0 h, 24 h, 48 h, and 72 h and used for oxidative stress analyses (overall reactive oxygen species (ROS), superoxide dismutase (SOD), and catalase).

#### 4.6.2. Overall Reactive Oxygen Species

The total ROS produced in this study was determined using 2′-7′dichlorofluorescin diacetate (DCFH-DA), a cell-permeable fluorescent dye that detects reactive oxygen species [49]. The interacted homogenized (using the method mentioned before) samples were combined with 100 µL of (100 µM) DCFH-DA and incubated in a dark environment for 30 min. The fluorescence intensity of the samples was measured using a spectrofluorometer (JASCO FP-8300, Tokyo, Japan). The wavelengths of excitation and emission were 485 nm and 530 nm, respectively. The results of the fluorescence spectrums of all treated samples were compared to those of the control samples.

#### 4.6.3. Superoxide Dismutase

The technique was used to assess the role of superoxide dismutase in tomato plants (leaf, shoot, and root) [50]. The capacity of superoxide dismutase to limit superoxide activity in nitro blue tetrazolium (NBT), which is generated by light-reduced riboflavin and oxygen, is the basis for this test. The interacted homogenized samples (using the method mentioned before) were centrifuged at 13,000 rpm for 20 min, and the supernatants were collected separately. A chemical solution that comprised 50 mM Na_2_CO_3_, 96 mM NBT, 20 mM hydroxylamine hydrochloride, and 0.6 percent Triton X-100 was added to 100 µL of each supernatant and incubated for 20 min in the UV zone at 37 °C. UV–visible spectroscopy at 560 nm was used to determine the total absorbance intensity of the samples (HITACHI, U-2910, Tokyo, Japan).

#### 4.6.4. Catalase

Yilancioglu [51] examined catalase enzyme activity in tomato leaf, shoots, and roots in 2014. The interacted homogenized samples (using the method mentioned before) were centrifuged at 13,000 rpm for 20 min, and the supernatants were collected separately. Each 100 µL supernatant was added to 2 mL of 10.8 mM H_2_O_2_ solution. For this experiment, the hardness of the phosphate buffer solution that contained H_2_O_2_ was utilized as a baseline template. UV–visible spectroscopy at 560 nm was used to determine the total absorbance intensity of the samples (HITACHI, U-2910, Tokyo, Japan).

#### 4.6.5. Statistics

GraphPad Prism (version 5.0) software was used to perform statistical analysis on all the data. The difference between the control and the other NP-interacted samples, as well as the difference between the Flu−PS (nanoplastic), HA (humic acid), and Flu−PS+HA combinations, was measured using GraphPad Prism. For statistical data comparisons, two-way ANOVA was used [46]. *p* values of less than <0.05 were deemed statistically significant. All data are the standard error of the mean (S.E.M.) of at least three independent experiments performed in triplicate.

## 5. Conclusions

Currently, research is focused on the phytotoxic effects of Flu−PS on higher plants, using tomato (*Solanum lycopersicum*) as a typical bioindicator. Based on the results of the study, eco-coronated Flu−PS and HA produced comparable seed germination rates, chlorophyll estimates, root and shoot lengths, and a significant reduction in oxidative stress when compared to Flu−PS. The results showed that Flu−PS-treated plants grew more slowly than HA-treated plants and the control plants. The plant growth rate was increased with the addition of HA to Flu−PS treatment, while nanoplastic toxicity was decreased.

Eco-coronas formed on Flu−PS at the seed surface may have reduced Flu−PS uptake by tomato seeds by enhancing particle aggregation. It was determined that the natural organic substance humic acid, found in agricultural soil, reduced the toxicity of nanoplastics, one of the most significant emerging agricultural contaminants in recent years. This study is likely to contribute to a better understanding of the interaction between nanoplastics and organic compounds found in agricultural soils.

## Figures and Tables

**Table 1:** The particle size and zeta potential of PS and PS+HA.

Contents	Flu−PS	Flu−PS+HA
DLS size	235.5 nm	325.9 nm
Zeta potential (mV)	89.7 mV	−96.6 mV

## Data Availability

Not applicable.
